# Supplementary material for: Remnant of the late Permian superplume that generated the Siberian Traps inferred from geomagnetic data
Source: Nat Commun. 2023 Mar 10;14:1311. doi: 10.1038/s41467-023-37053-3 (PMC10006221; doi:10.1038/s41467-023-37053-3)
Supplement: Supplementary file 1 — Supplementary Information [file 41467_2023_37053_MOESM1_ESM.pdf]

Supplementary Information for

Remnant of the late Permian superplume that generated the Siberian Traps inferred from geomagnetic data

Shiwen Li[1], Yabin Li[1], Yanhui Zhang[2], Zikun Zhou[1], Junhao Guo[1], Aihua Weng[1]*

[1] College of Geo-exploration Science and Technology, Jilin University, Changchun 130026, China;

[2] School of Safety Engineering and Emergency Management, Shijiazhuang Tiedao University, Shijiazhuang 050043, China.

Correspondence to: wengah@jlu.edu.cn (A. Weng)

This PDF file includes:

Supplementary Table. 1

Supplementary Figures. 1 to 10

Supplementary References

Supplementary Tables

Supplementary Table S1. Details of the observation stations used in this study. Latitude and longitude correspond to the geographic coordinates of the station, GM corresponds to the geomagnetic coordinates, and the data length is the duration of the recorded time-series of geomagnetic field data. (The geomagnetic field data at stations were downloaded from the World Data Centre: http://www.wdc.bgs.ac.uk/.)

Code	Station	Latitude	Longitude	GM Latitude	GM Longitude	Data Length
AAA	Alma Ata	76.92	43.18	34.31	152.94	1963-2015
ARS	Arti	58.57	56.43	49.2	140.05	1973-2018
ASH	Ashkhabad	58.11	37.95	31.06	135.29	1959-1990
BGY	Bar Gyora	35.09	31.72	28.27	112.66	1989-2018
BOX	Borok	38.23	58.07	53.45	123.61	1977-2020
ELT	Eilat	34.95	29.67	26.27	112.09	1998-2015
KGD	Bereznyaki	73.08	49.82	41.22	150.64	1965-1988
KIV	Kiev	30.3	50.72	47.58	113.54	1958-2019
KSH	Kashi	76.0	39.5	30.73	151.65	1995-2017
KZN	Kazan	48.85	55.83	49.8	131.71	1957-2016
MOS	Moscow	37.32	55.47	51.08	121.67	1957-2020
NVS	Novosibirsk	83.23	54.85	45.47	159.93	1966-2020
ODE	Odessa	30.88	46.78	43.67	112.69	1941-1991
SVD	Ekaterinburg	61.07	56.73	49.22	142.2	1930-1980
TFS	Tbilisi	44.7	42.09	36.96	123.99	1957-2001
TKT	Tashkent	69.62	41.33	33.13	146.22	1957-1998

Supplementary Figures

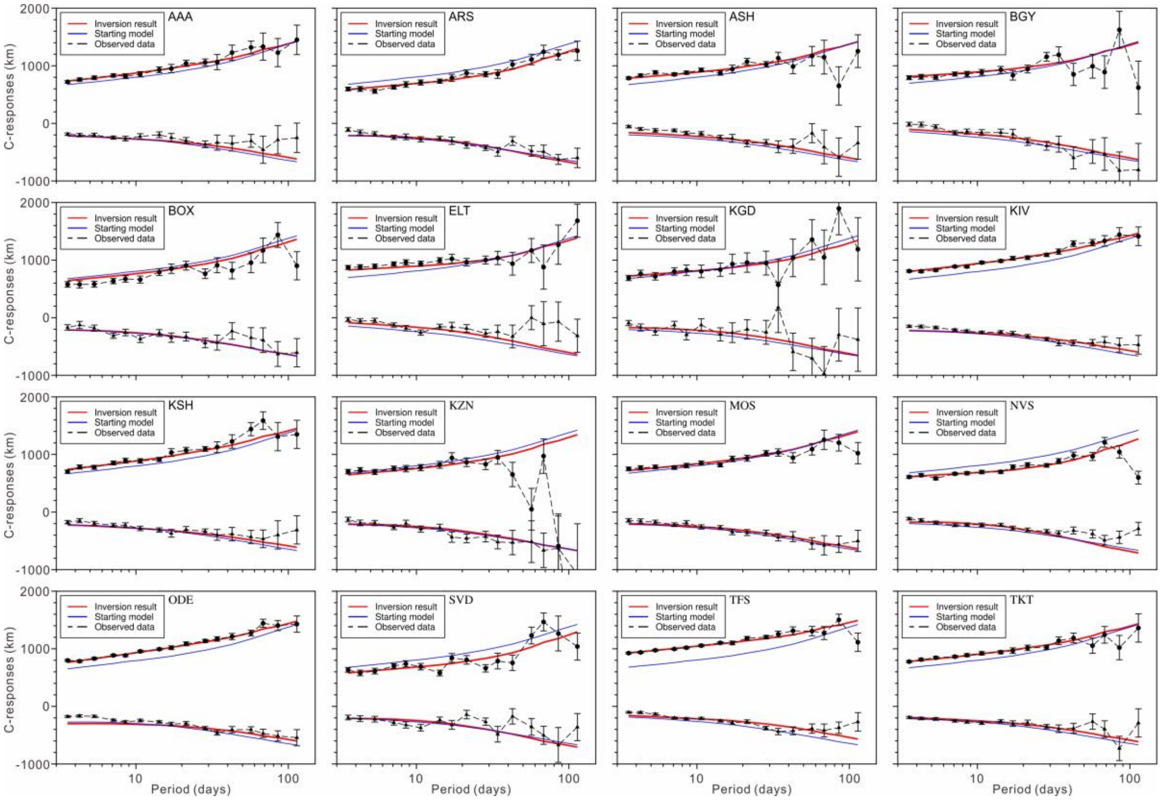

Supplementary Figure S1. C-responses and their associated data fits. The responses of the starting model[1] (solid blue lines), inversion result (solid red lines) and observed data (dashed black lines) are drawn for the stations used in this study. The vertical error bars represent the data misfit at each observed period.

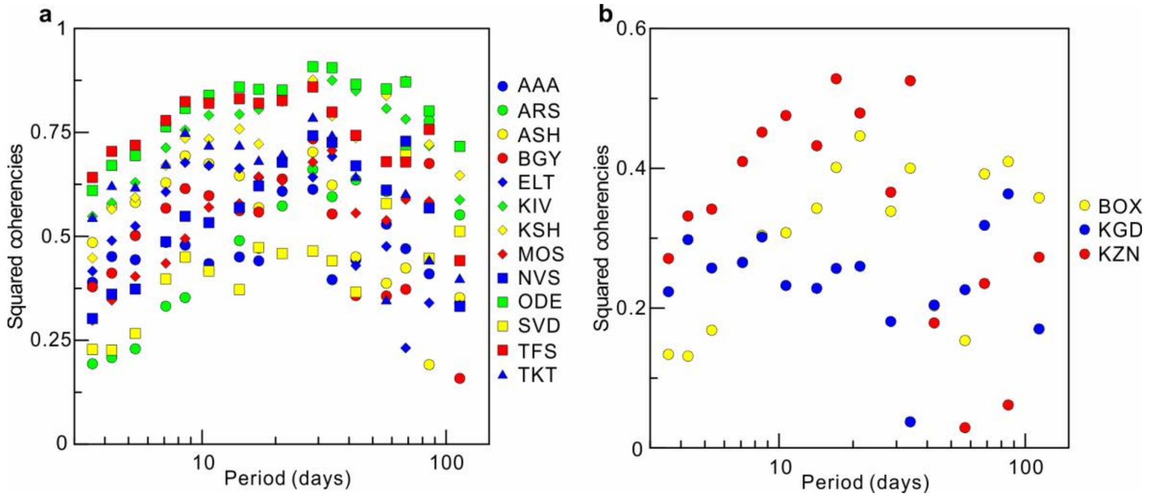

Supplementary Figure S2. Squared coherencies[2] of H_r (vertical component of the geomagnetic field) and H_θ (horizontal component of the geomagnetic field) at 16 selected stations. (a) and (b) correspond to the good- and poor-quality C-responses, respectively.

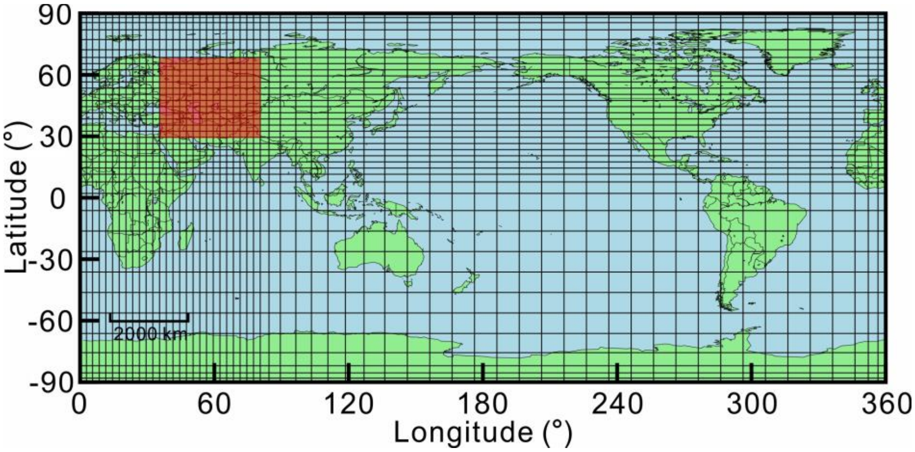

Supplementary Figure S3. Heterogeneous grid used for each shell in our inversions. The study area is outlined by the red rectangle.

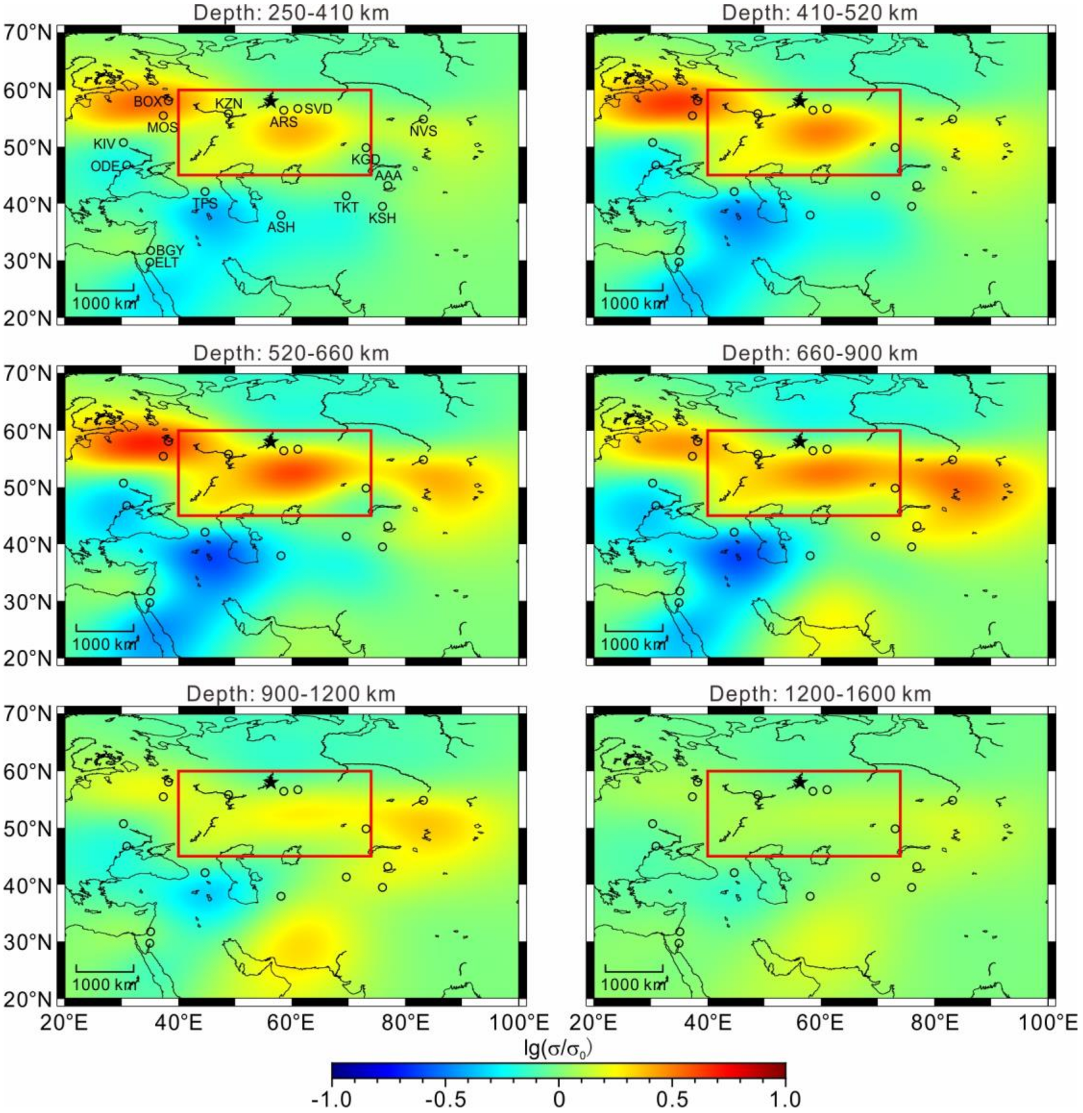

Supplementary Figure S4. Variations in electrical conductivity at depths of 250 to 1600 km. The red rectangle denotes the Perm electrical conductivity anomaly (PEC). The open circles indicate observatory locations used in geomagnetic depth sounding inversion. The star marks the location of Perm city.

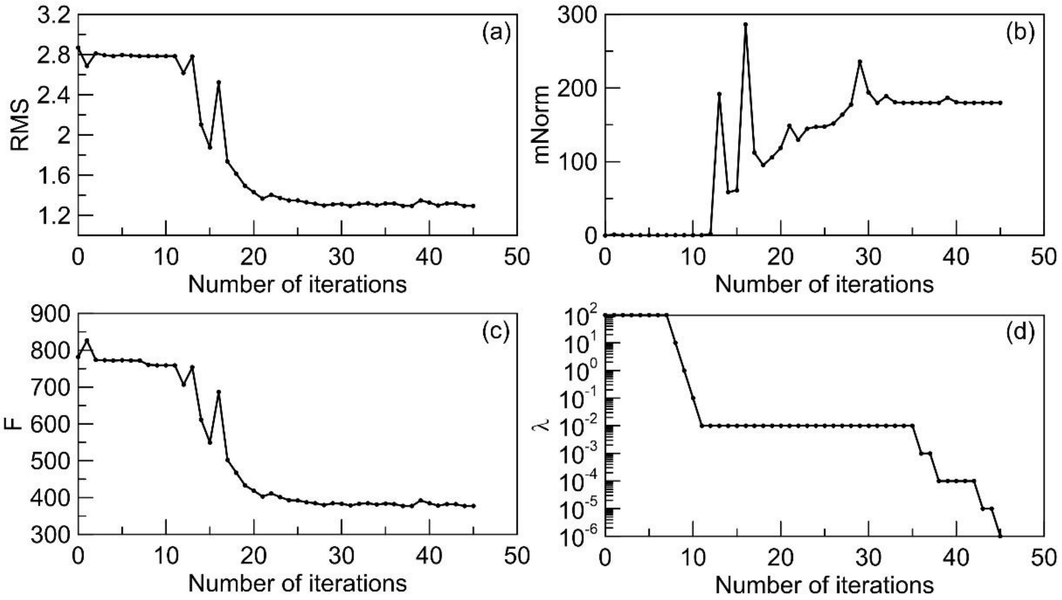

Supplementary Figure S5. Variations in the (a) data fitting error (shown as the RMS of the misfit), (b) model roughness (mNorm), (c) penalty function value (F), and (d) regularisation factor (λ) during inversion of the preferred model. The model corrections generally converge to constant values as the RMS of the misfit stabilises at a minimum value, which means that additional inversion iterations do not change the model conductivity. The reduction in the regularisation parameter with additional iterations cannot improve the RMS of the misfit, whereas the convergence of the RMS of the misfit means that the inversion has obtained an optimal data fit.

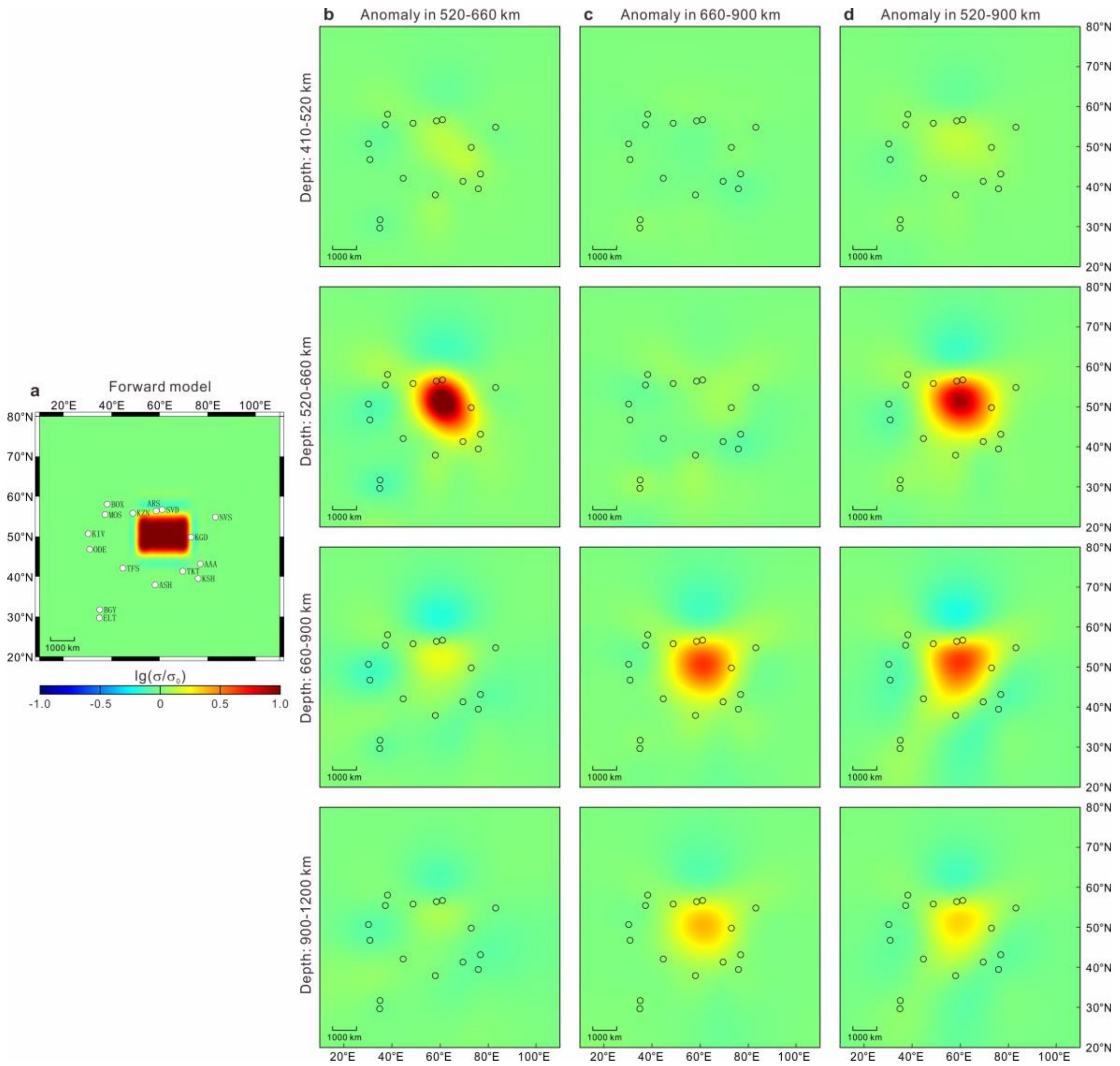

Supplementary Figure S6. Resolution tests. The observation distribution near the study area is used in the tests. (a) The forward model for tests, with a 20° × 10° (longitude × latitude) perturbation introduced to the conductivity field. This conductivity anomaly is ten times higher than that in the one-dimensional average model (background model). Inversion results are shown when the conductivity anomaly is placed in the (b) 520–660 km, (c) 660–900 km, and (d) 520–900 km depth intervals. The anomaly was well recovered at the appropriate depth range in each test.

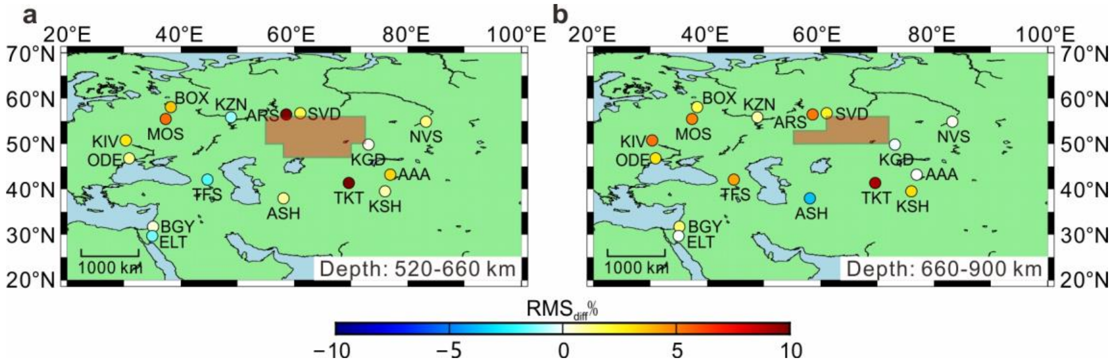

Supplementary Figure S7. Robustness tests for anomaly A. The conductivity of anomaly A (shaded red polygon) at the (a) 520–660 km and (b) 660–900 km depth is replaced by the conductivity of the initial model. The different colours at the stations indicate the changes (in percent) in the root mean square (RMS) of the data misfit error.

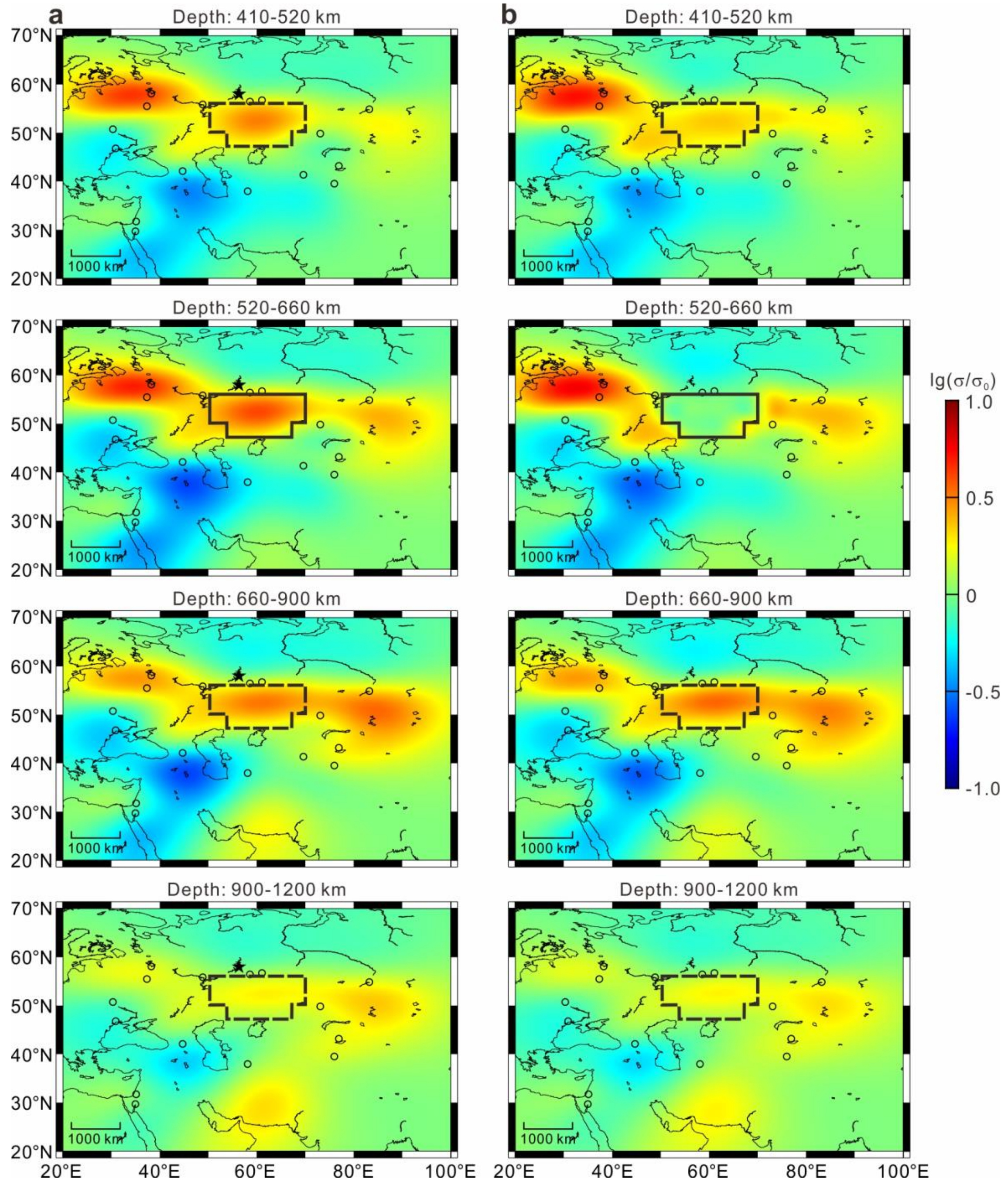

Supplementary Figure S8. Comparison of inversion results when the conductivity of the upper anomaly PEC (520–660 km depth) is varied. (a) Preferred inversion result. (b) Inversion result when the conductivity of the upper anomaly PEC (indicated by the black polygon) is set to the initial value and fixed in the inversion. The defects in the electrical structure within the polygon arise from the GMT interpolation strategy (https://www.generic-mapping-tools.org/).

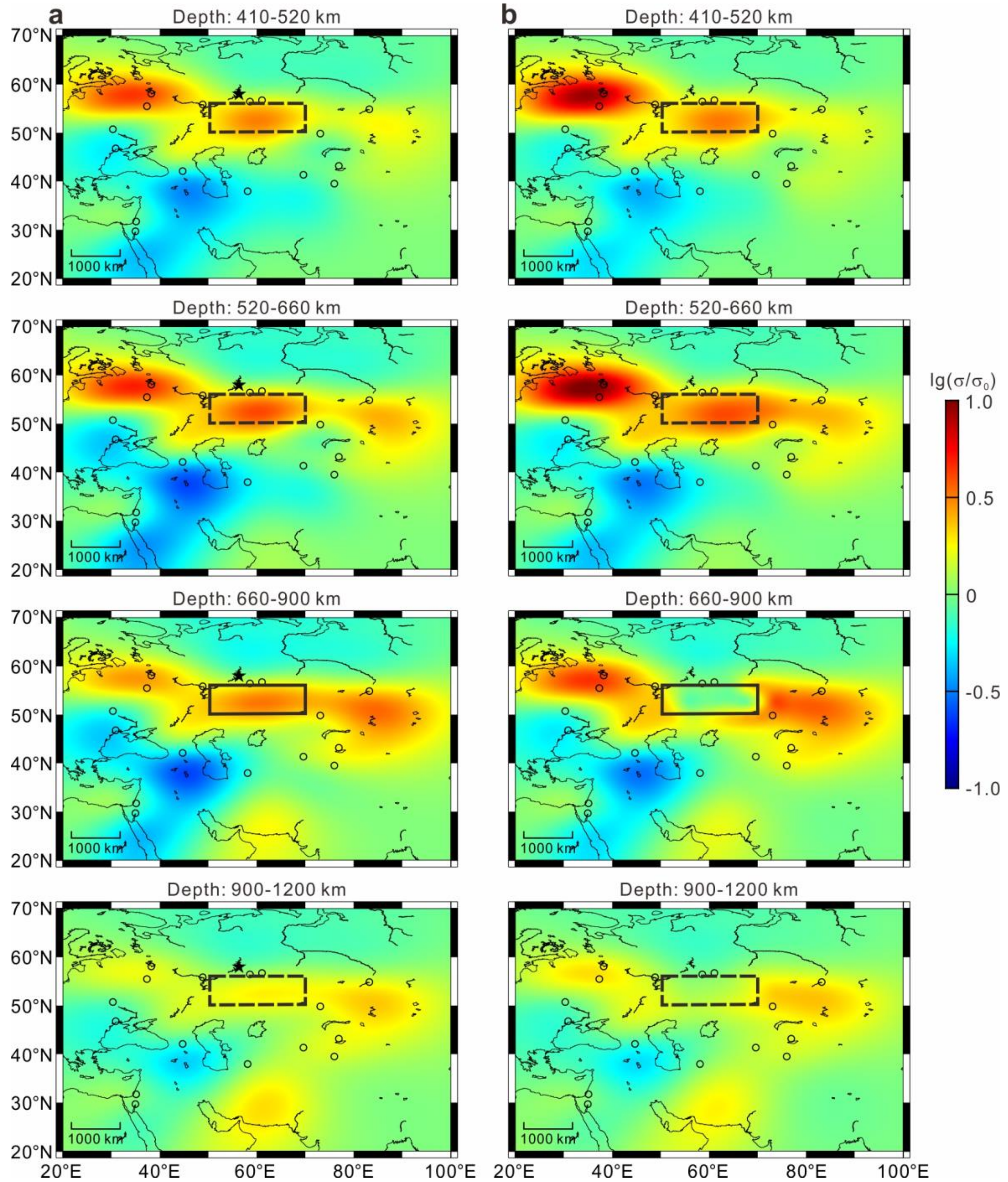

Supplementary Figure S9. Comparison of inversion results when the upper anomaly A (660–900 km depth) is varied. (a) Preferred inversion result. (b) Inversion result when the conductivity of the upper anomaly A (indicated by the black polygon) is set at the initial value and fixed in the inversion. The defects in the electrical structure within the polygon arise from the GMT interpolation strategy (https://www.generic-mapping-tools.org/).

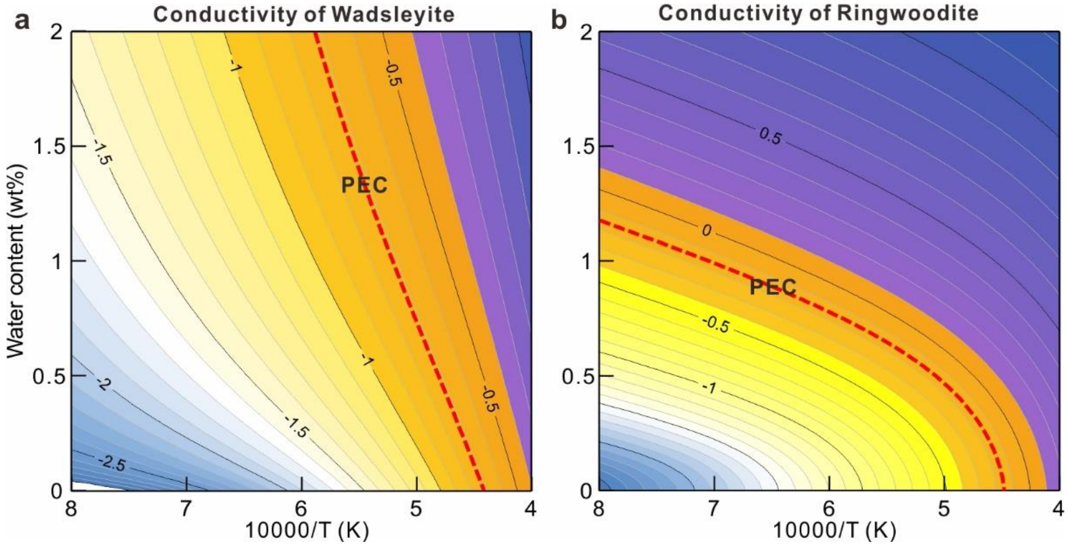

Supplementary Figure S10. Electrical conductivities of wadsleyite (a) and ringwoodite (b) as functions of temperature (T) and water content (in wt.%)[3]. The shaded red zones indicate the conductivity range in the PEC, and the dashed red lines denote the average conductivity of the PEC.

Supplementary References

1. Kelbert, A., Schultz, A. & Egbert, G. Global electromagnetic induction constraints on transition-zone water content variations. *Nature* **460**, 1003–1006 (2009).
2. Semenov, A. & Kuvshinov, A. Global 3-D imaging of mantle conductivity based on inversion of observatory C-responses-II. Data analysis and results. *Geophys. J. Int.* **191**, 965–992 (2012).
3. Yoshino, T., Manthilake, G., Matsuzaki, T. & Katsura, T. Dry mantle transition zone inferred from the conductivity of wadsleyite and ringwoodite. *Nature* **451**, 326–329 (2008).
